# Pedometers and Accelerometers in Multiple Sclerosis: Current and New Applications

**DOI:** 10.3390/ijerph191811839

**Published:** 2022-09-19

**Authors:** Jeffer Eidi Sasaki, Gabriel Felipe Arantes Bertochi, Joilson Meneguci, Robert W. Motl

**Affiliations:** 1 Graduate Program in Physical Education, Federal University of Triangulo Mineiro, Uberaba 38025-180, MG, Brazil; 2 Department of Kinesiology and Nutrition, College of Applied Health Sciences, University of Illinois Chicago, Chicago, IL 60612, USA

**Keywords:** physical activity, sedentary behavior, wearable technology, assessment, multiple sclerosis

## Abstract

Pedometers and accelerometers have become commonplace for the assessment of physical behaviors (e.g., physical activity and sedentary behavior) in multiple sclerosis (MS) research. Current common applications include the measurement of steps taken and the classification of physical activity intensity, as well as sedentary behavior, using cut-points methods. The existing knowledge and applications, coupled with technological advances, have spawned new opportunities for using those motion sensors in persons with MS, and these include the utilization of the data as biomarkers of disease severity and progression, perhaps in clinical practice. Herein, we discuss the current state of knowledge on the validity and applications of pedometers and accelerometers in MS, as well as new opportunities and strategies for the improved assessment of physical behaviors and disease progression, and consequently, personalized care.

## 1. Introduction

Wearable motion sensors, namely pedometers and accelerometers, have been instrumental in improving the assessment of physical behaviors in research studies, especially physical activity, sedentary behavior, and sleep. Of note, data collected with these devices have allowed for a better understanding of the associations between physical activity and health outcomes in different populations [1]. Within the field of multiple sclerosis (MS), the use of wearable motion sensors for measuring physical activity behavior has advanced significantly over the last two decades [2].

The growing use of wearable motion sensors in MS is a result of physical activity being currently recognized as an essential therapeutic component [3], as it has been consistently associated with beneficial effects for MS symptoms and function [4,5]. Physical activity is traditionally defined as any bodily movement produced by the contraction of the skeletal muscles that results in increased energy expenditure [6], whereas sedentary behavior is defined as any waking activity performed in a seated, reclined, or lying position with an energy expenditure ≤1.5 MET [7]. To date, the majority of studies on motion sensors in MS have focused on examining the validity and/or reliability of pedometers and/or accelerometers in assessing steps taken and physical activity-related metrics, such as energy expenditure or time spent in sedentary behavior and different physical activity intensities [8]. These applications have allowed for significant progress in understanding how device-measured physical activities and sedentary behaviors are related with health in persons with MS. However, there are major opportunities and possibilities for further applying wearable motion sensors and exploring the resulting data for identifying signatures representing free-living physical function and mobility status in MS.

To date, some research groups have examined wearable motion sensors data in MS as digital biomarkers for detecting and monitoring disease progression and severity, especially mobility impairments [9]. The use of digital biomarkers in general is relatively incipient, and further research can contribute toward identifying common ground for their best use in MS. This application may help researchers and clinicians in identifying mobility and physical function deterioration in persons with MS, based on ecologically-valid data [8], thereby allowing for timely physical behavior interventions for attenuating disease progression and slowing the physical disability process.

**Figure 1:**
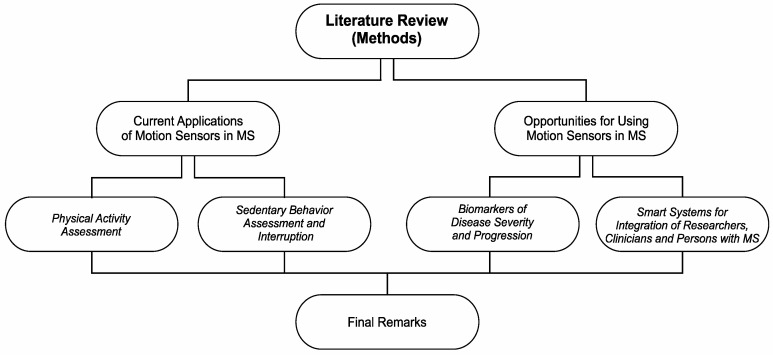
Manuscript structure.

Based on such a perspective, we believe that it is timely and imperative to understand the current applications of wearable motion sensors, particularly pedometers and accelerometers in MS, and the next steps for advancing the field. We adopted a narrative review, defined as a type of review to discuss specific topics/themes from a theoretical and contextual point of view [10], in order to (a) provide a brief overview on the current use of wearable motion sensors for assessing physical activity and sedentary behavior in MS, and to (b) discuss new opportunities for using motion sensor data as digital biomarkers for disease severity and progression in MS based on physical function outcomes. To accomplish these objectives, we structured this manuscript according to the sections and subsections depicted in Figure 1.

## 2. Methods

We conducted a structured, non-exhaustive search of the literature. The literature search was conducted in PubMed using the following search strategy: ((((((((((((((accelerometer) OR (accelerometry)) OR (pedometer)) OR (pedometry)) OR (“motion sensor”)) OR (“activity monitor”)) OR (wearable)) AND (“physical activity”)) OR (“motor activity”)) OR (“sedentary behavior”)) OR (“Walking”)) OR (“physical function”)) OR (“physical disability”)) OR (“mobility disability”)) AND (“multiple sclerosis”). The search returned 2925 articles. We then applied the following criteria for narrowing the number of articles: (1) original or review studies; (2) the population of interest was MS; (3) the research examined the validity and/or reliability of motion sensors, or proposed new methods and/or techniques for analyzing data from motion sensors, or even verified patterns of accelerometry data based on the degree of physical disability; and (4) the outcome of interest was physical activity, sedentary behavior, gait, physical function, or physical/functional disability. Two of the authors (JES and GFAB) evaluated the titles and abstracts of the articles, and identified 71 of them that met the above criteria. Of this total, we selected 23 articles [2,5,8,9,11,12,13,14,15,16,17,18,19,20,21,22,23,24,25,26,27,28,29] that were deemed the most relevant for constructing the arguments and discussions within the focus of this narrative review. The other 35 studies cited in this review were selected as being supportive of our arguments and points of view. These additional studies did not necessarily include MS as a population of interest. The strategy adopted to write the review was a narrative format, as it provided us with more flexibility to present our ideas and points of view on the topic.

## 3. Current Applications of Motion Sensors in MS

### 3.1. Physical Activity Assessment

The majority of the current wearable motion sensors are of small size, can be placed on different body parts, and use piezoelectric, piezoresistive, or capacitive sensing mechanisms [1]. The most commonly used wearable motion sensors in MS have been pedometers and accelerometer-based activity monitors [8]. These devices allow for the collection of different measures related to physical activity, such as number of steps, step-rate, kcals/min, raw acceleration (g-force), and activity counts. Studies have tested the validity and reliability of these devices in MS and have developed physical activity prediction methods specifically for this group of people.

Overall, most pedometers have been demonstrated to be accurate in recording steps during regular and fast walking speeds in MS [11,12]. Regarding slow walking speeds (e.g., ≤54 m/min), piezoelectric- or accelerometer-based pedometers are more accurate than spring-levered pedometers [13,30]. Of note, accuracy of pedometers in persons with MS depends on the disability level, with a higher measurement error occurring for those with severe MS disability, as demonstrated by Sandroff et al. [14], where the accuracy of the ActiGraph GT3X activity monitor decreased from 95.5% to 87.3% relative to manually counted steps. Conversely, the StepWatch, which is a gold-standard device for capturing steps in clinical populations, only demonstrated a very small decrement in accuracy during slow walking speeds in persons with severe MS disability (99% to 95.7%, relative to manually counted steps) [14]. Such results denote that the StepWatch may better differentiate the mobility status in free-living situations than the ActiGraph GT3X if step is the metric of interest. The high accuracy of the StepWatch establishes it as a reference method that can be used to validate other pedometers under free-living conditions [8], since there is a lack of such devices that have truly undergone ecological validity testing.

In the last few years, there has been a “boom” of fitness/activity trackers in the market. These devices have further been applied to assess steps taken in MS [31], and have presented moderate to high accuracies in recording steps when compared to manually counted steps (manually counted vs. Fitbit—ICC = 0.69; manually counted vs. Fitbit One—relative accuracy = 98.1%) [15,16]. One major problem of using these devices in research concerns the lack of transparency by the manufacturers on how the outputs are derived/calculated [8,32]. This turns the metrics from Fitness/Activity Trackers into a blackbox [8,32]. In order for these devices to have better acceptance among researchers, manufacturers would need to consider disclosing the proprietary predictive equations/algorithms in the devices. This seems very unlikely, as the proprietary predictive equations/algorithms along with software and device features are major differentiators among brands. Thus, many commercial interests are involved in this context and the companies are more likely to pursue the general consumer as opposed to the researcher costumer, considering the market size.

Regarding accelerometry, researchers have traditionally applied cut-points to classify physical activity intensity in MS [8]. This method involves the translation of accelerometer output into meaningful metrics of physical activity, a process termed calibration, wherein researchers establish the relationship between accelerometer output (i.e., counts/minute) and energy expenditure (i.e., metabolic equivalents (METs)) [1,33]. Within the field of MS, several disturbances in physical function, including altered gait parameters (e.g., walking velocity, cadence, step length, and stride length), result in a higher oxygen cost (O_2_ cost) during ambulation, compared with the general population [17,34], and this manifests as higher MET values per a given number of activity counts per minute. To address this issue, two studies [18,19] have established accelerometer cut-points for fully ambulatory persons with MS, and another study [17] has derived cut-points for persons with MS according to disability status. Accounting for the disability status is important because it identifies differences in the relationship of accelerometer output with oxygen cost for the activities, and further because as disability increases, locomotion speed tends to decrease. As demonstrated by Sandroff et al. [17], for individuals with mild disability, slow, comfortable, and fast walking speeds were 2.22, 2.84, and 3.21 mph, whereas for individuals with severe disability, the speeds for the same categories were 0.97, 1.40, and 1.74 mph, respectively. Thus, it becomes clear that one should not apply the same cut-points for individuals with different disability statuses, as the functional capacity is highly different, meaning that the cut-points for individuals with mild MS disability will be too high for individuals with severe MS disability. This indicates that researchers should not apply cut-points developed for the general population among individuals with MS. The MS-specific cut-points have been important for improving the understanding of associations between physical activity and different health outcomes in MS [35], both in observational and experimental studies [36,37,38,39].

Recently, the cut-points approach has been applied to pedometer output, wherein step-rate thresholds are established for physical activity intensity classification [8]. The advantage of using step-rate for predicting physical activity intensity relies on the fact that: firstly, step is an easy-to-understand metric; secondly, measures of steps from different pedometers are relatively comparable; and thirdly, most accelerometers currently provide the number of steps beyond activity counts as an output. This suggests that the same step-rate cut-points approach may be applied across brands and models of pedometers and accelerometers, thereby allowing for greater comparability across studies. Two studies have proposed step-rate cut-points for classifying moderate and vigorous physical activity in MS [20,21]. Agiovlasitis [20] has reported that the step-rate cut-points for moderate and vigorous physical activity were 99 and 144 steps/min, respectively, for persons with MS who had minimal walking impairment, and 96 and 136 steps/min for those with mild– moderate MS walking impairment. The other study [21] generated step-rate cut-points accounting for a broader range of MS-related ambulatory disability. The cut-points for moderate- and vigorous-intensity physical activity based on disability status were: (a) mild disability: 99 and 170 steps/min; (b) moderate disability: 89 and 160 steps/min; and (c) severe disability: 79 and 150 steps/min.

These step-rate cut-points provide researchers with a simple off-the-shelf method for assessing the time spent in moderate-to-vigorous physical activity in MS. As an analogous output across different pedometers and accelerometers, step-rate is a promising metric for a further examination of the associations of physical activity with health, function, and disability in research studies in MS. The step-rate cut-points further facilitate exercise prescriptions for lay individuals, as steps/min consists of an easy-to-understand metric of exercise intensity.

The abovementioned methods have been decisive for progress in the field; however, these methods have not caught up with the overwhelming advances in motion sensor hardware technology. For example, many of the current motion sensors collect high-resolution data, but limited attempts have been made to develop, validate, and operationalize machine-learning algorithms for activity classification in MS. Among the general population, such algorithms have mostly been developed and tested in the laboratory, demonstrating promising results for classifying a variety of activities, including the activities of daily living [40,41]. Within MS, the identification of activity type could be of major importance for monitoring locomotion and daily function, and thereby for providing an indication of disease severity and progression. Researchers in our field should further explore this application. In a later section of this paper, we highlight why monitoring certain features of free-living physical activity consists of a major opportunity for applying motion sensors in MS.

### 3.2. Sedentary Behavior Assessment and Interruption

Sedentary behavior assessment in MS has gained attention because of its negative associations with health outcomes [42]. Accelerometers allow for capturing the total time spent on sedentary behavior, and further, the number of breaks in such behavior, as well as the durations of these breaks. Some current motion sensors and prediction algorithms allow for the detection of postural transitions (e.g., sit-to-stand or vice versa), and this may provide an indication of lower body function under free-living conditions [22].

Studies using accelerometers suggest that persons with MS with mobility disability spend 65% (8.9 h/day) of their daily time in sedentary behavior, compared with 60% (8.4 h/day) for those without mobility disability [23]. The number of bouts in sedentary behavior lasting more than 30 min was slightly higher in persons with MS with mobility disability compared with those without mobility disability (5.1 vs. 4.3 bouts, *p* = 0.02) [23]. Accelerometer data further indicate that sedentary behavior is higher in older adults with MS compared with middle-aged and young adults with MS [43], and that longer durations of sedentary bouts partially correlate with lower physical and cognitive functions in older adults with MS [44]. These studies have been instrumental for establishing the deleterious associations of sedentary behavior with mobility disability and health outcomes in MS. However, the studies lack the power for inferring causal relationships. One next step in studies using accelerometers to examine sedentary behavior in MS is to apply such devices longitudinally for long periods of time, comprehending periods of relapses and/or disease worsening, allowing for a better understanding of how mobility disability, as well as physical and cognitive functions, relate to changes in sedentary behavior over time.

Some challenges for utilizing accelerometers for periods longer than 20 days are battery life, memory, the continuous upload of data to a cloud system, and especially participant burden. If these problems were overcome, then researchers would be able to hand the motion sensors to participants and keep contact remotely to check on the correct use and possible issues with the device. Currently, a protocol of continuous monitoring of individuals with MS for extended periods (20+ days) would involve periodic visits to the laboratory for data downloading and the delivery of a new, fully charged device to the participant. Participant burden also relates to the device placement, with most researchers still adopting the hip as the placement of choice. Nevertheless, there is a tendency for a growing number of researchers to adopt wrist-worn accelerometry protocols, reducing participant burden and allowing for data collection over the 24 h cycle [32]. In addition to changing the accelerometer placement, the improvement of device size and design by manufacturers are a major aspect for reducing participant burden. Research-grade accelerometers are not as comfortable to use as consumer-grade accelerometers.

Besides only monitoring sedentary behavior, wearable motion sensors may be applied for promoting interruptions of prolonged sitting in MS. Some devices, such as the activPAL, already present buzzing features for alerting individuals about interrupting sitting at given intervals (e.g., hourly or every two hours). These prompting signals may help with examining the potential health benefits of interrupting sedentary time and replacing it with light or moderate physical activity [45,46]. Therefore, in addition to only monitoring physical behaviors, motion sensors may be used to reduce sedentary behavior and increase physical activity in MS.

**Figure 2:**
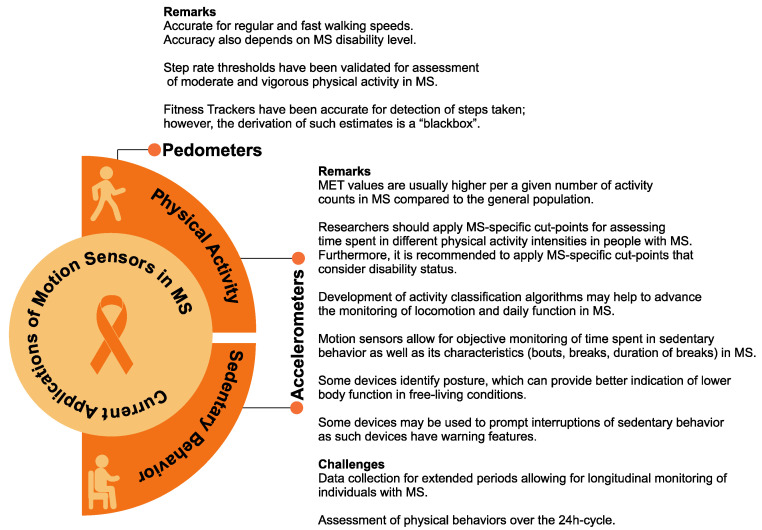
Summary of the evidence on the use of accelerometers and pedometers in MS.

A summary of the evidence described in Section 3.1 and Section 3.2 is provided in Figure 2.

## 4. Opportunities for Using Motion Sensors in MS

### 4.1. Biomarkers of Disease Severity and Progression

Overall, the field has witnessed substantial progress in using wearable motion sensors for assessing physical activity and sedentary behavior in MS. As the technology for the devices and data processing methods are evolving, new opportunities for assessing physical behaviors and clinical outcomes have emerged. Motion sensor data have the potential for tracking disease progression and severity based on the ecologically valid assessment of physical function and mobility [5]. Such an approach would bring additional information to existing laboratory-based tests.

For example, researchers and clinicians have typically adopted performance tests to assess physical function and mobility in MS [24]. However, there is evidence that performance-based tests may not present ecological validity [47]—these tests and outcomes may not totally reflect free-living performance, which entails unsupervised monitoring in the wild [48]. Conversely, wearable sensor technology in MS allows for the collection of free-living data that may serve as biomarkers of disease severity and progression. The continuous monitoring of physical activity with motion sensors allows for real-world data on mobility variables, such as walking speed, distance, and patterns, as well as postural transitions (e.g., sit-to-stand and stand-to-sit), to be obtained [24,49,50].

There is evidence that free-living accelerometer data may be related to performances on different mobility tests. Studies have indicated that ActiGraph 7164 activity counts were strongly correlated with walking speed (r = 0.82) [25] and mobility measures, such as the 6 min walk test (ρ = 0.78), and the Timed Up and Go test (ρ = −0.68) [26]. Similarly, pedometer output (step counts) has been correlated with measures of walking performance, mobility, and disability over a 7-day period (EDSS (ρ = −0.90), Multiple Sclerosis Walking Scale-12 (MSWS-12; ρ = −0.83), Timed 25-Foot Walk (ρ = −0.64), Timed Up and Go test (ρ = −0.51), and 6 min walk test (ρ = 0.67)) [27].

Those results provided a proof-of-concept that free-living accelerometer data may be related to physical function, and recent evidence suggests that unsupervised assessments are necessary for acquiring real-world mobility data [48,51]. This is especially true, because gait characteristics from shorter walking bouts during daily living appear to be more informative about the disability level than longer bouts that are typically applied in laboratory-based tests [28]. This reinforces the need to further collect motion sensor data in the free-living setting. With evolving technology, it may be possible in the future to collect data continuously without the need for researchers to only collect data at selected periods.

Despite the limited number of studies in this emerging area, some promising results have indicated that steps/day, accelerometer counts, and/or minutes/day in moderate-to-vigorous physical activity, decline with the progression of the disease in MS [23]. The effects of, and recovery from a relapse, in MS have been detected based on steps/day data assessed with motion sensors [29,52]. One recent study has demonstrated that data from triaxial accelerometers placed on the thigh and chest were correlated with clinically relevant measures, and differentiated non-fallers from fallers (accuracy rate: 74%) [53]. These results suggest that motion sensors and the range of output appear clinically relevant for monitoring disease progression and activity in MS, potentially representing the signature measures of walking, mobility, and disability.

Technological advances have resulted in the development of sophisticated Inertial Measurement Units (IMUs); these devices combine accelerometers, gyroscopes, and magnetometers. The use of IMUs in MS is timely because it may allow for the early detection of gait impairment, a hallmark of MS, based on an assessment of gait parameters (e.g., speed, step length, stride length, step time, swing time, and stance time) [24]. There is robust evidence that some gait parameters assessed with IMUs are significantly correlated with EDSS and MSWS-12 scores in MS (EDSS × Speed, r = 0.60; EDSS × Step Length, r = 0.46; EDSS × Step Time, r = 0.32; MSWS-12 × Speed, r = 0.64; MSWS-12 × Step Length = 0.37) [24], denoting the potential of these devices in providing important information on disease severity and progression, perhaps in daily life. An aspect of major potential is the assessment of gait smoothness, which has been examined in previous studies [54], and has detected early alterations in walking, in people with MS without disability. Pau et al. [54] studied the trunk accelerations of 50 people with MS without disability (EDSS = 1), and 50 age-matched healthy controls. Gait smoothness was assessed using the Harmonic Ratio (HR), and the results have indicated that people with MS presented lower gait smoothness than healthy controls (2.92 vs. 3.67, *p* < 0.001). Therefore, motion sensors data may be used for the early detection of decrements in the quality of walking.

Activity recognition and gait parameters assessments based on accelerometer and IMUs data could enable the consolidation of real-world mobility and physical function metrics as biomarkers of disease severity and progression in MS [24,50]. Over the past decade, important progress in activity recognition using accelerometer data has been made in general, especially with the high-sampling capabilities of currently available accelerometers (e.g., a sampling rate of 100 Hz) [1]. Laboratory studies have developed activity recognition algorithms that accurately classify several types of activities using data from waist- and wrist-worn accelerometers [40,41,55,56]. Activities successfully recognized using these algorithms include locomotion, activities of daily living, sport activities, postures, and transitions.

These algorithms could have important applications in MS, especially by providing information on specific activities (e.g., walking and sit-to-stand transitions) performed by persons with MS in real-world settings. Some incipient algorithms using wavelet transformation have already been applied to assess gait characteristics and parameters in MS [28]. The results clearly indicated that wearable motion sensors data are promising biomarkers of disease progression and severity [28]. Thus, the appropriate use of such data could allow for better self-management in MS, as well as effective and timely interventions by researchers and clinicians via integrated smart systems.

### 4.2. Smart Systems for the Integration of Researchers, Clinicians, and Persons with MS

Smart systems for the ongoing monitoring of physical behavior among those with MS are essential for establishing and using biomarkers for the early detection of disease progression, and consequently, for effective and timely interventions for disease management [57]. These smart systems could record real-world accelerometer and IMUs data continuously, with minimal burden for the end-users [8]. Additionally, smart systems should meet the demands of researchers, clinicians, and end-users (persons with MS), and allow for a more integrated and inclusive disease management approach.

In a clinical perspective, the continuous monitoring of free-living walking and physical functions may provide clinicians with important ecological information on individuals who need immediate attention to prevent the worsening of their mobility status, as well as their disease progression. Conversely, researchers need data on the continuous monitoring of free-living physical behavior in order to develop more accurate methods/algorithms for predicting mobility and physical function outcomes, as well as disease progression. This can in turn be used by health professionals to improve clinical practice and disease management in MS by improving decision making, time of decisions, and strategies for mitigating or reversing deterioration in mobility, physical function, and disease course.

**Figure 3:**
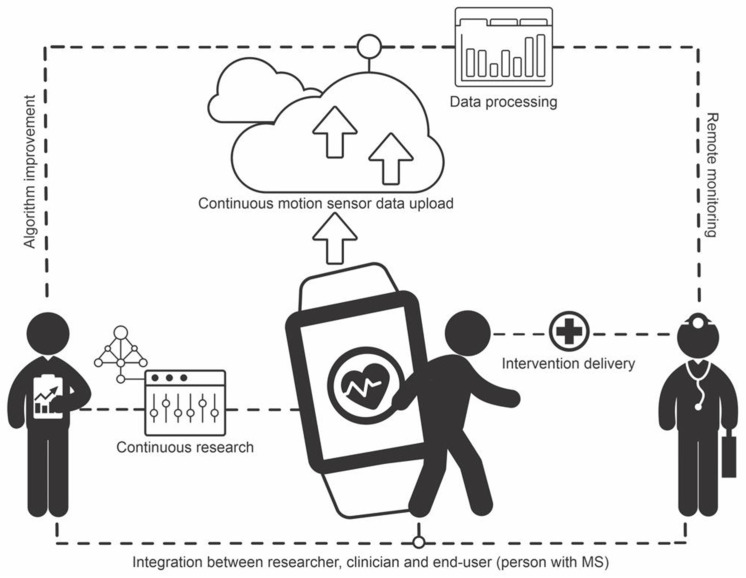
Illustration of the structure of a smart system for integration of researchers, clinicians, and people with multiple sclerosis. End-user motion sensor data are continuously uploaded to the cloud and processed via algorithms embedded in the cloud. Researchers use the data for algorithm improvement. Clinicians monitor patients remotely and deliver interventions as needed.

The integration of researchers, clinicians, and MS may be accomplished with digital cloud storage and computing technology, whereby motion sensor data for persons with MS are constantly collected, uploaded to the cloud, and processed remotely via algorithms implemented in servers, with outcomes and alerts being automatically sent to researchers and clinicians. Researchers may use the data to continuously improve the algorithms, whereas clinicians may use the data to improve clinical practice, providing personalized medicine to patients. Ultimately, this circle of constant feedback leads to better care for those with MS. Figure 3 illustrates the structure of such a system.

Beyond assessing real-world walking, physical function, and disease progression, smart systems may be valuable in the delivery and monitoring of behavioral interventions in MS. One review study highlighted the potential of using mobile platforms/systems for exercise interventions in persons with mobility disability [58]. In this regard, motion sensor data may be utilized in these mobile platforms/systems for mobile detection, biofeedback, and data processing and analysis, and therefore improve rehabilitation for persons with MS who have mobility restrictions. Such platforms/systems will permit superior reach and continuous physical behavior change interventions, which can ultimately lead to meaningful benefits for mobility, physical function, and disease progression in MS.

## 5. Conclusions

This manuscript summarized the current applications of wearable motion sensors, namely pedometers and accelerometers, in assessing physical activity and sedentary behaviors in MS, and presented evidence for the validity of commonly used devices, as well as available methods for processing data. Some drawbacks were identified for these existing methods, and we introduced ideas to aid such deficiencies. We further presented opportunities for advancing the field forward by using wearable motion sensors, including activity recognition algorithms, and the utilization of IMUs for assessing gait parameters. We lastly highlighted opportunities pertaining to the development of smart systems for the continuous monitoring of physical behaviors in MS, and thereby obtaining real-world data that may serve as biomarkers of disease progression. These smart systems can promote the integration of researchers, professionals, and persons living with MS, and allow for the timely and ongoing detection of disease progression, and timely interventions.

Researchers should consider the evidence presented herein when conducting future investigations. There are many aspects of pedometry and accelerometry that may be improved for obtaining more accurate physical activity estimates, or even for obtaining different physical activity variables, as discussed in the different sections of this manuscript. We hope that the evidence and ideas postulated here may help with advancing the field forward.

## Data Availability

Not applicable.
